# Long-Term Efficacy of Mepolizumab at 3 Years in Patients with Severe Asthma: Comparison with Clinical Trials and Super Responders

**DOI:** 10.3390/biomedicines11092424

**Published:** 2023-08-30

**Authors:** Diego Bagnasco, Stefania Nicola, Elisa Testino, Luisa Brussino, Laura Pini, Marco Caminati, Federica Piccardo, Rikki Frank Canevari, Laura Melissari, Alessandro Ioppi, Luca Guastini, Carlo Lombardi, Manlio Milanese, Francesca Losa, Michela Robbiano, Laura De Ferrari, Anna Maria Riccio, Giuseppe Guida, Marco Bonavia, Donatella Fini, Francesco Balbi, Cristiano Caruso, Pierluigi Paggiaro, Francesco Blasi, Enrico Heffler, Giovanni Paoletti, Giorgio Walter Canonica, Gianenrico Senna, Giovanni Passalacqua, on behalf of SANI

**Affiliations:** 1 Allergy and Respiratory Diseases, IRCCS Policlinico San Martino, University of Genoa, 16132 Genoa, Italyrobbianomichela@gmail.com (M.R.); laura231283@hotmail.it (L.D.F.); passalacqua@unige.it (G.P.); 2 Department of Internal Medicine (DIMI), University of Genoa, 16132 Genoa, Italy; f.piccardo4@gmail.com; 3 SCDU Immunologia e Allergologia, AO Ordine Mauriziano di Torino, C.so Re Umberto 109, 10128 Torino, Italyluisa.brussino@unito.it (L.B.); 4 Respiratory Medicine Unit, ASST—“Spedali Civili” of Brescia, Piazzale Spedali Civili 1, 25123 Brescia, Italy; laura.pini@unibs.it; 5 Department of Medicine, University of Verona, 37134 Verona, Italy; ma.caminati@gmail.com (M.C.); gianenrico.senna@univr.it (G.S.); 6 ENT Department, IRCCS Policlinico San Martino, University of Genoa, 16132 Genoa, Italy; canevari@edu.unige.it (R.F.C.); luca.guastini@unige.it (L.G.); 7 Departmental Unit of Allergology, Immunology & Pulmonary Diseases, Fondazione Poliambulanza, 25124 Brescia, Italy; carlo.lombardi@poliambulanza.it; 8 Department of Respiratory Diseases, S. Corona Hospital, ASL2, 17027 Pietra Ligure, Italy; manlio.milanese@unige.it; 9 UO Allergology and Clinical Immunology, ASST Mantova, 46100 Mantova, Italy; francesca.losa@asst-mantova.it; 10 Department of Clinical and Biological Science, University of Torino, 10043 Orbassano, Italy; giuseppe.alesgui@gmail.com; 11 Department of Rehabilitation Pulmonology, Hospital Ge-Arenzano, ASL3, 16149 Genoa, Italy; marco.bonavia@asl3.liguria.it; 12 Department of Pneumologiy, Hospital Sarzana (SP), 19125 La Spezia, Italy; donatella.fini@asl5.liguria.it; 13 Department of Pneumologiy, Hospital Imperia, 18100 Imperia, Italy; balbifrancesco@libero.it; 14 Department of di Medical and Surgical Science, Fondation Universitary Policlinic A. Gemelli IRCCS, University Cattolica Sacro Cuore, 20123 Rome, Italy; carusocristiano1@gmail.com; 15 Department of Surgery, Medicine, Molecular Biology and Critical Care, University of Pisa, 56126 Pisa, Italy; pierluigi.paggiaro@unipi.it; 16 Department of Pathophysiology and Transplantation, Università degli Studi di Milano, 20122 Milan, Italy; 17 Respiratory Unit and Adult Cystic Fibrosis Center, Internal Medicine Department, Fondation IRCCS Ca’ Granda-Ospedale Maggiore Policlinico, 20122 Milan, Italy; 18 Unit of Personalized Medicine, Asthma and Allergy, IRCCS Humanitas Clinical and Research Hospital, 20089 Rozzano, Italy; enrico.heffler@hunimed.eu (E.H.); giovanni.paoletti@hunimed.eu (G.P.); canonica.gw@gmail.com (G.W.C.); 19 Department of Biomedical Sciences, Humanitas University, 20072 Pieve Emanuele, Italy

**Keywords:** severe asthma, eosinophils, mepolizumab, CRSwNP, IL-5, real life, registry

## Abstract

The efficacy mepolizumab in severe asthmatic patients is proven in the literature. Primarily to study the effect of mepolizumab on exacerbations, steroid dependence, and the continuation of efficacy in the long term. Secondarily to evaluate the effect of the drug on nasal polyps. Analyzing data from SANI (Severe Asthma Network Italy) clinics, we observed severe asthmatic patients treated with mepolizumab 100 mg/4 weeks, for a period of 3 years. 157 patients were observed. Exacerbations were reduced from the first year (−84.6%) and progressively to 90 and 95% in the second and third ones. Steroid-dependent patients decreased from 54% to 21% and subsequently to 11% in the second year and 6% in the third year. Patients with concomitant nasal polyps, assessed by SNOT-22, showed a 49% reduction in value from baseline to the third year. The study demonstrated the long-term efficacy of mepolizumab in a real-life setting.

## 1. Introduction

Asthma is a chronic airway disease characterized by bronchial obstruction, usually reversible either spontaneously or with therapy. It affects over 300 million people worldwide, representing one of the highest prevalence diseases in the field of respiratory diseases [1]. The severe form of this disease affects 5–10% of all patients and is characterized by poor symptom control despite maximal inhaled therapy dose, recurrent exacerbations and frequent use of systemic oral corticosteroids (OCS) to gain symptom control [2]. To perform real precision and personalized medicine, it started with a phenotyping of patients and continued with an endotyping linked to inflammation characteristics and cellularity [1]. The more detailed understanding of the mechanisms of inflammation facilitated and promoted the process of patients’ endotyping, allowing for the identification of identify new biological therapeutic options for patients with severe forms of disease. Beginning with the role of immunoglobulin (Ig) E, then continuing with the one of eosinophils and fractional exhaled nitric oxide (FeNO), and finally with the deepest knowledge of cytokines and proteins, like interleukin (IL) 4, 5, 13, 23, 33, and thymic stromal lymphoid protein (TSLP), cells like Innate lymphoid (ILC) type 2, and T helper lymphocyte (TH) 2, a deepest knowledge of disease inflammation mechanisms was allowed [3,4]. In relation to the type of inflammation, two different mechanisms were identified: the first one related to type 2 (T2) inflammation, and the second, not associated with T2 cells and cytokines, that still remains a challenge [5,6], and is actually not easily treatable with marketed biologics. T2 inflammation generally shows a good response to corticosteroids, the use of which, however, implies short, and long-term side effects [7,8].

With the aim of searching for a control of disease, limiting systemic corticosteroid use, and reducing exacerbations, several biologic drugs were developed [9]. One of the main targets of these drugs are eosinophils, which are observed to be cells that are usually increased in the blood of severe asthmatic patients. Among biologics, mepolizumab (MEP) was the first to be marketed for patients with an eosinophilic endotype of the disease [10]. MEP is a monoclonal antibody against IL-5 that controls eosinophils’ proliferation, maturation, and activity [11,12]. The link between IL-5 and eosinophils is well known in fact, this cytokine is necessary for the maturation of their precursors located in the bone marrow and subsequently for the release of mature cells into the blood [13]. The production and stimulation of IL-5 are prompted by TH2 and ILC2 cells, the latter being in turn principally activated by signals from epithelial cytokines like IL-25, IL-33, and TSLP [14,15].

The effects of MEP were studied first in randomized controlled trials (RCT) [16,17,18] and then in real-life studies [19,20,21], confirming its efficacy and providing useful information on some aspects such as its pharmacoeconomics and the effect on comorbidities [22,23,24,25]. The effect of MEP was clearly demonstrated in patients with eosinophilic severe asthma with a cell count greater than 300/μL in the 12 months prior to the drug’s administration and at least 150 at the time of the first dose. Clinical trials focused on the efficacy of the medication in a sample of patients generally treated for 24–52 weeks, and recent extension studies demonstrated the efficacy after several years. A recent real-life (RL) study demonstrated the drug’s efficacy in a sample of 51 patients observed for 36 months [19].

RL studies can provide additional and complementary information to RCTs [26], especially on long-term effects and safety. This is essential in the case of biological drugs, where the populations of regulatory trials and RL often differ. Long-term efficacy of MEP was addressed only in clinical trials and in a maximum of 2 years of real-life observations; with this manuscript, we want to describe data from 3 years of analysis on asthmatic patients treated with MEP. Considering that chronic rhinosinusitis with nasal polyposis (CRSwNP) is the more frequent comorbidity in severe asthma, we also evaluated the effects of MEP in such selected patients, from clinic afferents to the national Italian severe asthma registry (SANI).

## 2. Methods

**Figure 1:**
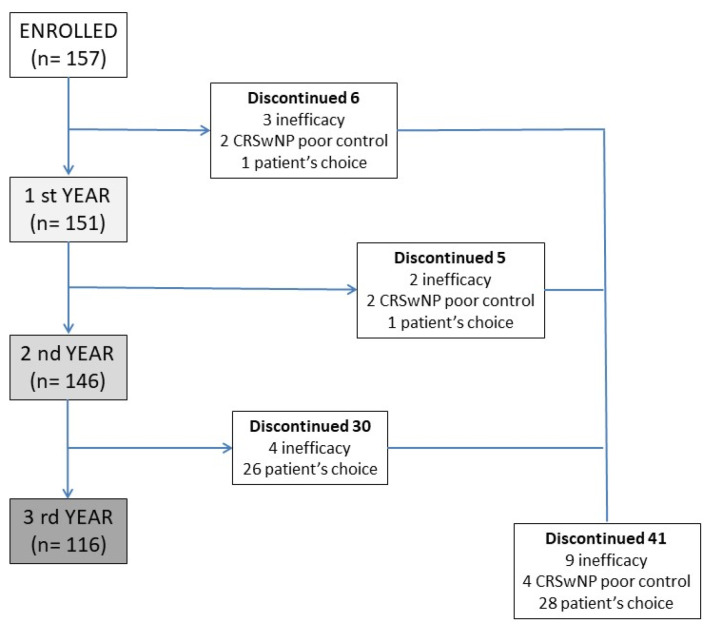
Patients’ disposition during the 3 years of observation, with discontinuation rates and the reason of drug suspension. CRSwNP—Chronic rhinosinusitis with nasal polyps.

A prospective multicenter observational study was developed, involving several severe asthma centers in the SANI (Severe Asthma Network Italy) registry [27], with the aim of analyzing the data from patients treated, for severe asthma with MEP 100 mcg subcutaneously/4 weeks for at least 3 years. No restriction about the age of patients was chosen. Patients analyzed were all affected by T2 inflammation, confirmed by blood eosinophil counts. All patients were eligible for treatment with MEP according to the prescribing criteria of the Italian regulatory agency (uncontrolled severe asthma, eosinophils > 150 cells/mcL at the time of first administration and >300 in the previous 12 months, at least 2 exacerbations in the previous 12 months, systemic steroid therapy lasting for more than 6 months). The diagnosis of asthma was done according to the reversibility of obstruction or methacholine test. The first administration was given between June 2017 and January 2019, and this warranted 3 years of observation. The reasons for which some patients discontinued the MEP treatment were carefully recorded and analyzed. All patients were evaluated for exacerbations and use of OCS (converted to prednisone equivalent). Lung function tests were performed at baseline and every 12 months (inhaled therapy was discontinued one half-life before the test), as well as the Asthma Control Test (ACT) and Fractional exhaled nitric oxide (FeNO). All patients were evaluated for chronic rhinosinusitis with endoscopic tests and/or CT-scan imaging, and the clinical impact of nasal polyps was assessed with the Sinonasal Outcome Test (SNOT-22), which was performed at baseline and every 12 months. The disposition of patients is summarized in Figure 1. This study used the data from patients included in the SANI registry; therefore, all of them provided signed consent to use their data for medical research, previously approved by Genoa’s ethics committee (year 2017, ID 3663). All data were analyzed with descriptive statistics. An indirect comparison was made with the so-called super responder patient cohort [28].

The appropriate statistical analysis was applied according to the characteristics of the variables in the exam. Fisher’s exact test, χ^2^, one-way ANOVA, and Student *t* test were used when necessary.

## 3. Results

At the beginning of the study were enrolled 157 patients, 51% male, with a mean age of 59 (range 21–84). Of them, 99 patients (63%) also had CRSwNP. Eosinophils mean level before starting treatment was 718 (±579) cells/μL; 85 (54%) of patients were steroid-dependent with a mean dose of administered prednisone of 15 (±11) mg/day and 5.8 g/year. Mean exacerbations rate at baseline was 3.9 ± 2.8 with 1.4 (±0.5) exacerbations. The mean value of FEV1 was 2.21 ± 1.0 L corresponding to 70 ± 33% of the predicted value. The control of the disease was evaluated with ACT (17 ± 4 at baseline). Patients with CRSwNP had a SNOT-22 score of 51 ± 15 (Table 1).

MEP reduced exacerbations as early as the first year, with a decrease from 3.9 ± 2.8/year to 0.6 ± 1.2/year (F = 58.8; *p* < 0.0001). The reduction continued in subsequent years, with a mean of 0.4 ± 0.9/year and 0.2 ± 0.5/year in the second and third years of observation. No statistical difference was observed between the reduction of exacerbations in the years following the first, highlighting the maintained and prolonged efficacy of the drug over time.

**Figure 2:**
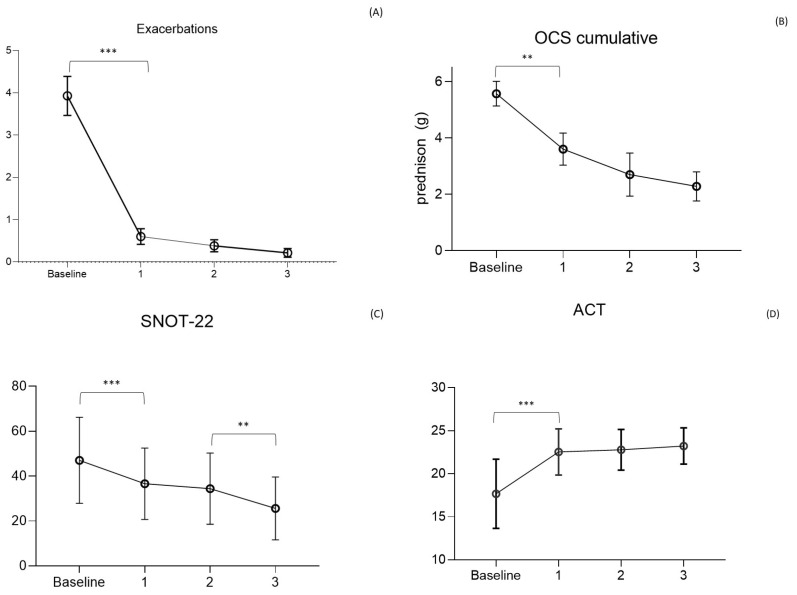
Main outcomes of the study. (**A**) Asthma exacerbations rate per year (Mean ± SD); (**B**) Oral corticosteroids (OCS) cumulative dose/y (g prednisolone) in steroid dependent patients; (**C**) Asthma Control Test (ACT) score; (**D**) Sinonasal Outcome Test (SNOT)-22 score (mean ± SD). *** *p* < 0.01; ** *p* < 0.05.

OCS-dependent patients decreased between the beginning of the study and the first year, going from 54% to 21% (*p* < 0.0001), with a progressive reduction as low as 11% (*p* = 0.02) and 6%, respectively, at the second and third years. The mean daily dose of OCS in dependent patients decreased from 15 ± 11 mg of prednisone at baseline to 9.8 ± 10 mg at the first year (F = 0.57; *p* = 0.022), 7.6 ± 9.0 mg at the second, and 6.3 mg/day at the third year. The calculated cumulative dose decreased from 5.8 ± 4.0 g of prednisone-equivalent per year at baseline to 3.6 ± 3.1 g the first year and 2.7 ± 2.7 g and 2.1 ± 1.3 g, respectively, in the second and third years. Over 3 years of observation, the drug allowed the complete discontinuation of steroids in 87% of dependent patients (summing those who discontinued the drug and those who maintained MEP administration), being able to reduce the average daily dose to 6.3 ± 4 mg (Figure 2) in the remaining OCS dependent patients. Lung function tests, measured using FEV1, demonstrate an increase in the value of 170 mL in the first year, overcoming the minimal clinically important difference (MCID) value of 100 mL [29] and keeping it stable in the three years of observation.

Asthma control, as per ACT, showed an increase from 17 ± 4 at baseline to 23 ± 2 at the first year of observation (F = 27.16; *p* < 0.0001) when the value plateaued for the following two years. As for asthma, the control of nasal symptoms was also evaluated, with the SNOT-22 test showing a reduction from a baseline level of 51 ± 15 to 37 ± 15 (F = 0.32; *p* = 0.0002) in the first year and 34 ± 16 (*p* = 0.856) and 26 ± 14 (*p* = 0.05) respectively in the second and third years. The global main results, about asthma exacerbations, OCS cumulative dose, ACT, and SNOT-22 values, are condensed in Figure 2.

Patients who discontinued the therapy over the three-year observation period were 41 (26%), none of them for drug-related adverse events. Discontinuation due to drug inefficacy was recorded in 9 patients (6%), of whom only 4 required systemic steroid therapy with a mean of 5 mg per day. Of the remaining 32 patients, 4 interrupted therapy for inefficacy in nasal symptoms and frequent CRSwNP acute exacerbations, and 28 patients independently decided to suspend the therapy (Figure 1). The patients who requested discontinuation of the drug were all found to be controlled; the main reason for the request for discontinuation was their desire to suspend monthly biologic drug administration given full disease control.

The comparison of our patients with the cohort of the so-called “super responders” described by Kavanagh showed a statistically significant difference in BMI, where RL asthmatics were found to be thinner (25.8 ± 8.8 vs. 8.2 ± 4.5; *p* = 0.0008) but did not differ in exacerbations at baseline (3.9 ± 2.8 vs. 3.57 ± 2.2; *p* = 0.142), FEV1 measured in liters (2.21 ± 1.0 vs. 2.10 ± 0.65; *p* = 0.171), or in the concomitant presence of CRSwNP (63% vs. 67.9%; *p* = 0.188) (Table 2).

## 4. Discussion

**Figure 3:**
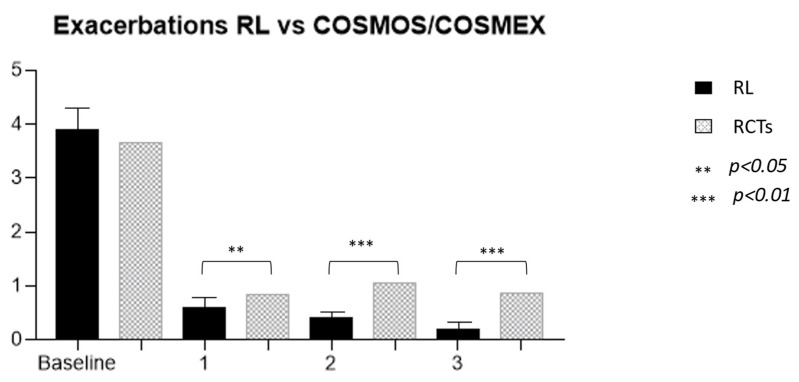
Comparison of exacerbation rates between our real-life cohort and Cosmos/Cosmex long-term trials.

The efficacy of MEP was clearly demonstrated both in RCTs [16,17,30,31] and in RL settings [32]. The prolonged administration of this drug allowed for the observation of its effects in patients treated for longer periods than the limited timeframe of RCTs, which was generally 24–52 weeks. The first observation that can be made from the present data analysis is that not only is the effect of the drug maintained over time but also that the reduction in exacerbation occurrence is progressive with time (Figure 2). These RL data confirm the one found in the extension trials of MEP (COSMEX), which showed a progressive and then maintained reduction of exacerbation rate in treated patients, as well as in those who were previously treated with placebo in the double-blind phase and after that with MEP in the unblinded one [33]. Even in real life, therefore, prolonged disease control is confirmed, which does not vary over time, providing the clinician with a prolonged expectation of efficacy in patients being treated with MEP for asthma. In addition, as already shown in other observational trials, real-life data results are more encouraging than in RCTs [34]. In fact, the cohort of treated patients we observed had a higher reduction in exacerbation rate over years in comparison to the COSMEX/COSMOS trials [33,35] (Figure 3). It is interesting to point out that, although the starting population of the two samples did not have a statistically different mean number of exacerbations (3.9 RL vs. 3.67 RCTs), in later years the reduction was more pronounced and continued to be progressively more pronounced in patients in RL (0.6 vs. 0.85, *p* = 0.012; 0.4 vs. 1.05, *p* < 0.0001; 0.2 vs. 0.86, *p* < 0.0001). The difference found between drug efficacy in real life and in RCTs, in this case COSMOS and COSMEX, could be related to the characteristics of the patients treated with MEP in RL. One of the first aims of biological therapies in asthma is certainly to reduce, or better yet, to discontinue, systemic steroid therapy. If we compare the average prednisone dose in OCS-dependent subjects of our cohort with the one reported by other works [17,36,37,38,39], our patients result in receiving a higher dose of systemic steroids at baseline. The first important observation regards the reduction of steroid-dependent patients, decreasing from 54% at baseline to 6% after 3 years of treatment. Secondarily, the mean daily intake of prednisone equivalent by each patient was reduced by 15 ± 11 to 6.3 ± 4 mg after 3 years. A more precise observation about long-term steroid side effects regards cumulative doses, expressed in grams, allowing to more specifically count not only the intake of dependent patients but also the one of people who use steroids only during exacerbations [8]. In our study, the mean dose of steroids corresponds to a cumulative dose of 5.8 ± 4 g per year. In the study, the dose could be reduced by 38% during the first year in steroid-dependent subjects (21% of the entire cohort compared to baseline), and then continued to be reduced in subsequent years up to a cumulative dose of 2.2 ± 1.3 g/year, in 6% of the cohort. The response to therapy remains constant over time with regard to the effect on exacerbations and respiratory function, already visible in the first year, while the effect on steroid dependence, already significantly reduced after 12 months of therapy, not only persists but progressively improves over the years, reaching discontinuation of OCS in 87% of patients after 3 years. The response to the drug in the observed cohort seems to be even better, compared with that described in randomized clinical trials. In particular, there was a significantly greater reduction in exacerbations since the first year of treatment, and this persisted throughout the duration of the study.

The efficacy of the drug on the RL cohort, particularly in exacerbation, OCS sparing effect, and disease control, appears to be similar to what is observed in super-responder patients, as described by Kavanagh, and better than what is described in the COSMOS study. Analyzing the characteristics of patients in RL, those in clinical trials, and those defined as super responders, focusing on differences and similarities, we found that a simultaneous presence of CRSwNP and a baseline better respiratory function were present in patients in whom we observed a better response to the drug. Both characteristics are present in our cohort and in the one described by Kavanagh. The presence of both factors, CRSwNP and more conserved respiratory function, seems to be factors able to influence a better response to the drug. Furthermore, the percentage of patients affected by asthma and CRSwNP too turns out to be higher than past publications; in the current analysis, it is 63% vs. 38–40% [40,41].

In addition to being an indicative marker of good response to therapy, CRSwNP also turns out to be a possible target of the drug. Thus, we described what happens to nasal symptoms reported by patients using the SNOT-22 questionnaire in those who have asthma and CRSwNP. What emerged is in line with data from dedicated registry clinical trials for patients with only CRSwNP. The reduction of the mean SNOT-22 values after one year of administration was higher than the minimal clinically important difference for the test, which is set to be 9 points by several authors [42] and 8.9 by others [43]. The impact of the drug not only on asthma but also on CRSwNP turns out to be successful, both from a clinical and pharmacoeconomic point of view. Patients with both of these conditions are known to be most frequently burdened by exacerbations, higher systemic corticosteroid use [32], and consequently higher acute and chronic OCS damage, as well as higher health care expenditures [44].

Our observation in real life would suggest that the definition of super-responder overestimates what happens in real life.

Lastly, the data on respiratory function, although not reaching statistical significance but far exceeding the MCID value, is not only important for the reported outcome of the patient, but in agreement with what other authors have described [45], a marked improvement in FEV1 can be considered an indirect sign of airway remodeling, adding an important feature to this drug.

In conclusion, MEP has been shown to be effective, also in RL and over a long observation period of 3 years, in reducing exacerbations and controlling asthma. The efficacy of MEP was also demonstrated in patients with CRSwNP comorbidity, which is far more prevalent in RL than in RCTs. The effect of the drug also appeared significant on CRSwNP itself, as evidenced by a progressive reduction in SNOT-22 values over the years. According to the recent literature, it can be hypothesized that the treatment, although effective, should be maintained for life [46].

## Figures and Tables

**Table 1:** Characteristics of patients at each year of observation and respective comparisons.

	Baseline (n = 157)	1° Year (n = 151)	*p*-Value Baseline vs. 1° y	2° Year (n = 146)	*p*-Value 1° vs. 2° y	3° Year (n = 116)	*p*-Value 2° vs. 3° y	*p*-Value Baseline vs. 3°
Male (%)	80 (51)	76 (50)	0.864	77 (53)	0.706	59 (51)	0.833	0.937
Age mean (range)	59 (21–84)	59 (22–85)	0.945	59 (23–81)	0.899	60 (24–82)	0.910	0.904
Age onset	41 (15.7)	n.a.	-	n.a.	-	n.a.	-	-
BMI	25.8 (8.8)	26.1 (7.6)	0.894	25.9 (6.4)	0.903	26.0 (6.1)	0.945	0.849
CRSwNP (%)	99 (63)	95 (63)	0.901	88 (60)	0.811	70 (60)	>0.999	0.705
Blood Eosinophils ^+^	718 (579)	88 (43)	<0.0001	91 (23)	>0.999	90 (31)	>0.999	<0.0001
OCS dependent (%)	85 (54)	31 (21)	<0.0001	16 (11)	0.02	7 (6)	0.19	<0.001
OCS daily dose °	15.0 (11)	9.8 (10)	0.022	7.6 (9)	0.891	6.3 (4)	0.933	0.046
OCS cumulative yearly dose (g)	5.8 (4.0)	3.6 (3.11)	0.039	2.7 (2.7)	0.867	2.2 (1.3)	0.822	0.049
Exacerbations	3.9 (2.8)	0.6 (1.2)	<0.0001	0.4 (0.9)	0.656	0.2 (0.5)	0.842	<0.0001
Hospitalizated patients (%)	35 (22)	2 (1.3)	<0.0001	1 (0.7)	>0.999	1 (0.8)	>0.999	<0.0001
Hospitalization ^§^	1.4 (0.5)	0.02 (0.18)	<0.0001	0.006 (0.08)	>0.999	0.03 (0.2)	>0.999	<0.0001
FEV1 %	70 (33)	83 (24)	0.158	82 (22)	0.981	84 (20)	0.933	0.206
FEV1 L	2.21 (1.0)	2.38 ^¥^ (1.0)	0.044	2.33 (0.86)	>0.999	2.39 (0.9)	>0.999	0.078
FeNO	58 (42)	34 (18)	<0.0001	38 (14)	0.443	35 (18)	0.718	<0.0001
ACT	17 (4)	23 (2) ^¥^	<0.0001	23 (2)	0.898	23 (2)	0.691	<0.0001
SNOT-22	51 (15)	37 (15) ^¥^	0.0002	34 (16)	0.857	26 (14)	0.05	<0.0001

All data are expressed as mean and SD, where not otherwise specified. ^+^ Eosinophils are expressed in cells/mcl. ^§^ mean of hospitalized, due to asthma, patients; ° in OCS-dependent patients, expressed as mg of prednisone equivalent. ^¥^ MCID (Minimal clinically important difference) value exceeded. BMI—Body Mass Index; CRSwNP—Chronic rhinosinusitis with nasal polyps; OCS—Oral corticosteroids; FeNO—Fractioned exhaled nitric oxide; ACT—Asthma Control Test; SNOT-22—Sino nasal outcome test.

**Table 2:** Comparison of the principal super responders characteristics, according to Kavanagh, between the RL cohort, Cosmos and Kavanagh patients.

RL vs. COSMEX/COSMOS	RL Cohort	Cosmos 651	*p*-Value	Kavanagh 28	*p*-Value (Cosmos vs. Kavanagh)	*p*-Value (RL vs. Kavanagh)
Exacerbations *	3.9 (2.8)	3.67	0.305	3.57 (2.2)	0.811	0.142
OCS dose °	15 (11)	12.5	<0.005	10	0.013	<0.0001
BMI	25.8 (8.8)	28.1 (6.1)	0.001	28.2 (4.5)	0.907	0.0008
FEV1 (L)	2.21 (1.0)	1.99 (0.7)	0.008	2.10 (0.65)	0.378	0.171
CRSwNP ^§^	99 (63%)	24 (7%)	<0.0001	19 (67.9)	<0.001	0.188

* *t*-test one sample, ° patients from Sirius; ^§^ z-test one proportion. BMI—Body Mass Index; CRSwNP—Chronic rhinosinusitis with nasal polyps; OCS—Oral corticosteroids; ACT—Asthma Control Test.

## Data Availability

Data are unavailable due to privacy.

## Abbreviations

ACT	Asthma Control Test
BMI	Body mass index
CRSwNP	Chronic Rhinosinusitis with nasal polyps
FeNO	Fractional exhaled nitric oxide
FEV1	Forced expiratory volume 1 s
Ig	Immunoglobulin
IL	Interleukin
MEP	Mepolizumab
OCS	Oral Corticosteroids
MCID	Minimal clinically important difference
RCT	Randomized controlled trials
RL	Real Life
SANI	Severe Asthma Network Italy
SNOT-22	Sinonasal Outcome Test
T2	Type 2

## Appendix A

**SANI:** C Allegrini, A Antonelli, A Aruanno, E Bacci, B Beghè, M Bonini, M F Caiaffa, C Calabrese, G Camiciottoli, M Cavallini, F Chiecobianchi, M E Conte, A G Corsico, L Cosmi, M T Costantino, G Costanzo, M Crivellaro, S D’Alò, M D’Amato, A Detoraki, M C Di Proietto, N C Facciolongo, S Ferri, V Fierro, M P Foschino, M Latorre, L Macchia, M Montagni, E M Parazzini, R Parente, V Patella, G Pelaia, F Puggioni, L Ricciardi, E Ridolo, J Rolo, N Scichilone, G Scioscia, P Solidoro, G Varricchi, A Vianello, M R Yacoub, B Yang.
